# Epidemiology of Celiac Disease in Western Iran during 2019-2021

**DOI:** 10.1155/2024/1112812

**Published:** 2024-04-18

**Authors:** Maryam Janatolmakan, Mehdi Zobeiri, Shahab Rezaeian, Shima Rostami, Mehnosh Akbari, Alireza Khatony

**Affiliations:** ^1^Social Development and Health Promotion Research Center, Kermanshah University of Medical Sciences, Kermanshah, Iran; ^2^Student Research Committee, Kermanshah University of Medical Sciences, Kermanshah, Iran; ^3^Clinical Research Development Center, Imam Reza Hospital, Kermanshah University of Medical Sciences, Kermanshah, Iran; ^4^School of Health, Kermanshah University of Medical Sciences, Kermanshah, Iran; ^5^Infectious Diseases Research Center, Kermanshah University of Medical Sciences, Kermanshah, Iran

## Abstract

Celiac disease is a growing global public health concern. This epidemiological study is aimed at determining the prevalence of celiac disease in Kermanshah, Western Iran, from 2019 to 2021, as well as the frequency of gastrointestinal and nongastrointestinal manifestations associated with the disease. In this cross-sectional study, the medical records of all patients with a confirmed diagnosis of celiac disease between 2019 and 2021 were reviewed. The average population during the study period was 2,058,545. A researcher-developed checklist was used as the data collection tool, and descriptive statistics were employed for data analysis. During the study period, there were 113 patients diagnosed with celiac disease, with a mean age of 29.1 ± 16.6 years. The three-year prevalence of celiac disease was 5.49 (95% CI: 5.17-5.82) per 100,000 population. Among these patients, 70% (*n* = 78) was female. The most common gastrointestinal manifestations of the disease were abdominal pain (77.8%), constipation (59.3%), and diarrhea (54.9%). Iron-deficiency anemia (64.6%) and vitamin D3 deficiency (46.1%) were the most common nongastrointestinal manifestations. Growth retardation was observed in 39.0% of patients. This study demonstrated a higher prevalence of celiac disease in Kermanshah compared to global statistics. Given the association of celiac disease with other conditions such as diabetes, irritable bowel syndrome, growth retardation, and iron-deficiency anemia, healthcare providers should consider screening patients for celiac disease. Furthermore, community-based education is crucial in raising awareness about the significance of adhering to a proper diet and reducing wheat consumption.

## 1. Introduction

Celiac disease (CD) is a chronic autoimmune disorder that results from consuming gluten in genetically predisposed individuals [1]. It is a global problem with varying prevalence rates across continents and countries, and its incidence is increasing worldwide [2]. A systematic review and meta-analysis published in 2018 revealed that the global prevalence of CD ranges from 0.05% to 2.6% [3]. However, in countries like China, India, and Iran, the prevalence of the disease is higher due to the significant consumption of wheat [1].

According to a systematic review and meta-analysis published in 2017, the prevalence of CD in Iran was 2% and 3% in studies that used duodenal biopsy and serological tests, respectively [4]. CD can occur at any age [1, 5] and is primarily diagnosed through duodenal biopsy and antitransglutaminase antibody measurement [6]. However, only a limited number of patients are diagnosed [7]. CD is characterized by intestinal and extraintestinal manifestations. The most common intestinal manifestations include diarrhea, bloating, abdominal pain, constipation, and anorexia. The most common extraintestinal manifestations are iron-deficiency anemia, autoimmune diseases, fatigue, headache, psychiatric disorders, infertility, and osteoporosis [6–8]. Treatment for CD involves adhering to a gluten-free diet [9]. Evidence suggests that early detection of symptomatic patients and implementing a gluten-free diet improves symptoms and quality of life [10].

Given the importance of understanding the prevalence of CD for managing the disease and patients and the lack of prevalence data for CD in Kermanshah Province, this study is aimed at determining the prevalence of CD in Kermanshah Province from 2019 to 2021, as well as the frequency of gastrointestinal and nongastrointestinal manifestations of CD.

## 2. Methods

### 2.1. Study Site

**Figure 1:**
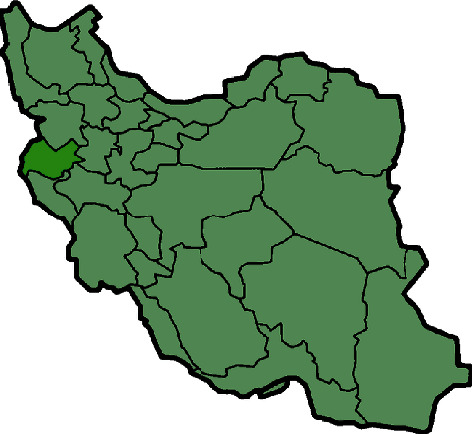
Iran map with Kermanshah Province highlighted. Source: https://commons.wikimedia.org/wiki/File:IranKermanshah.png.

This study was conducted in Kermanshah Province, a western province of Iran (Figure 1). The province comprises 14 cities and 84 villages, covering an area of 24,640 km^2^. According to the latest census, the population of Kermanshah province is estimated to be 2,164,656 people [11].

### 2.2. Study Population and Sampling

The study population included all patients covered by the celiac clinic affiliated with Kermanshah University of Medical Sciences who were included in the study using a census method. The inclusion criterion was having a medical record in the celiac clinic. This clinic serves as the main center for admitting and managing patients with CD in Western Iran and is staffed with a gastroenterologist and a nutritionist. The total number of CD cases identified from 2019 to 2021 was 113.

### 2.3. Study Design/Data Management

**Figure 2:**
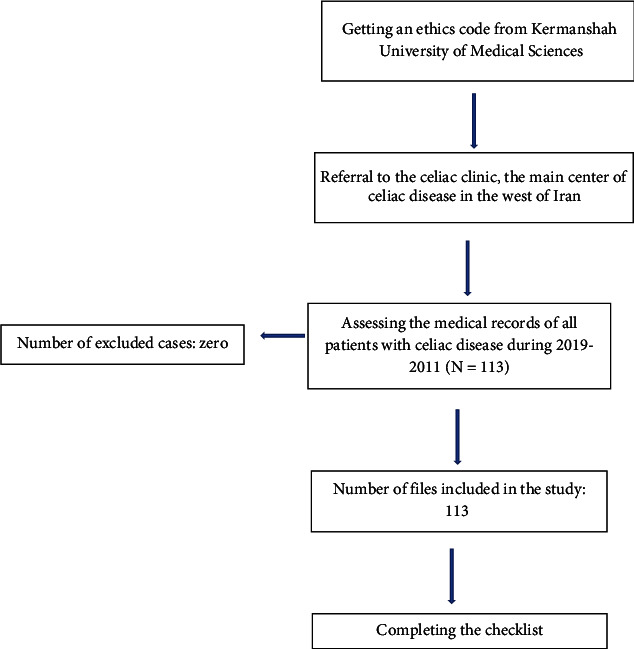
Flow diagram of the study.

This cross-sectional study was conducted based on the Strengthening the Reporting of Observational Studies in Epidemiology (STROBE) criteria [12]. The study instrument consisted of a two-part checklist. The first part included questions about patients' demographic information, including age, sex, education, occupation, body mass index (BMI), family history of CD, and family history of irritable bowel syndrome (IBS). The second part focused on the gastrointestinal and nongastrointestinal manifestations of CD. In adult patients (over 14 years), the diagnosis of the disease was established based on positive results from both serological tests and duodenal biopsies. However, in pediatric patients (under 14 years), the diagnosis was based solely on positive serological tests. In the current study, duodenal biopsy samples were assessed by a pathologist following the guidelines of Marsh's classification system [13], evaluating the presence of villous atrophy, crypt hyperplasia, and other histological features. Marsh stage 2 and above were considered positive. To collect the data, the researcher accessed the medical record section of the celiac clinic and documented the required information using a checklist. In cases where patients' medical records were incomplete, the researcher contacted them to obtain the necessary information (Figure 2).

In the current study, vitamin D deficiency was defined as follows: severe deficiency, serum 25(OH)D levels less than 25 nmol/L; moderate deficiency, between 25 and 50 nmol/L; insufficiency, between 50 and 75 nmol/L; and sufficiency, greater than 75 nmol/L [14]. The definition of IBS according to the Rome IV criteria is as follows: recurrent abdominal pain occurring at least one day per week for the past three months, accompanied by at least two of the following criteria: related to defecation, associated with a change in frequency of bowel movements and associated with a change in stool form [15]. The term “growth retardation” refers to a decrease in the rate of body growth and failure to achieve a reasonable physical size compared to the expected growth pattern based on age and gender [16].

The prevalence of CD was calculated by dividing the number of cases per year by the population of the province in that year. Descriptive statistics using SPSS 18 software were employed for data analysis, including frequency distribution indices, mean, and standard deviation.

### 2.4. Ethical Considerations

The study, with the code KUMS.REC 1399.1115, received approval from the ethics committee of Kermanshah University of Medical Sciences. Patients' details and information were treated confidentially and kept private.

## 3. Results

A total of 113 individuals with CD were included in this study. The mean age of the patients was 29.1 ± 16.6 years, and 78 (69%) of them were female. Among the patients under 14 years of age, the majority was female (*n* = 13, 56.5%), had a BMI less than 18.4 (*n* = 19, 82.6%), and had no family history of CD (*n* = 22, 95.6%) or IBS (*n* = 23, 100%). In the age group over 14 years, most patients were female (*n* = 65, 72.2%), unemployed (*n* = 81, 90%), had an undergraduate education (*n* = 71, 78.9%), had a normal BMI (*n* = 50, 55.5%), and had no family history of CD (*n* = 85, 94.4%) or IBS (*n* = 76, 84.4%) (Table 1).

The prevalence of CD in 2019, 2020, and 2021 was 1.33%, 3.25%, and 0.92%, respectively. The overall prevalence of CD was 5.49% per 100,000 population (95% CI: 5.17, 5.82) (see Table 2).

According to the type of symptoms, the manifestations of CD were categorized into two groups, gastrointestinal and nongastrointestinal, and were analyzed separately by age group (children under 14 years and adults over 14 years). In children, the most common gastrointestinal manifestations were abdominal pain (*n* = 15, 65.2%), bloating (*n* = 13, 56.5%), and constipation (*n* = 12, 52.7%). In adults, the most common gastrointestinal manifestations were abdominal pain (*n* = 73, 81.1%), diarrhea (*n* = 55, 61.1%), and bloating (*n* = 54, 60.0%) (Table 3).

Regarding nongastrointestinal manifestations, the results indicated that iron-deficiency anemia was the most prevalent manifestation among both children (*n* = 16, 69.6%) and adults (*n* = 57, 63.3%). Vitamin D3 deficiency was observed in 47.8% (*n* = 43) of adult patients and 39.3% (*n* = 9) of children. Diabetes mellitus was found in 11.5% (*n* = 13) of patients, with a higher prevalence in children (34.8%) than in adults (5.8%). Growth retardation was observed in 40.0% (*n* = 44) of patients, with a higher prevalence in adults (42.2%) than in children (26.1%). IBS was observed in 17.7% (*n* = 20) of patients, with a higher prevalence in adults (21.1%) than in children (4.4%) (Table 4).

## 4. Discussion

The current study found the prevalence of CD to be 5.49%. The results indicated a decrease in CD prevalence in 2021 compared to 2020 and 2019, which may be attributed to the COVID-19 pandemic and the decrease in patients visiting the celiac clinic. In a multilevel analysis conducted in 2021, the global prevalence of CD was estimated to be 0.77% (95% CI, 0.50% to 0.87%). In Asia, the prevalence of CD was reported to be 0.69%, with Saudi Arabia (1.42%) and Japan (0.05%) having the highest and lowest prevalence rates, respectively [1]. A systematic review and meta-analysis conducted in 2017 reported prevalence rates of 3% and 2% for CD in Iran based on studies that used serological tests and biopsies for diagnosis, respectively [4]. The high prevalence of CD in Kermanshah, Iran, as observed in this study, may be attributed to factors such as poor eating habits and high consumption of wheat. These factors can contribute to increased exposure to gluten, which is known to trigger CD in susceptible individuals.

The study found that the minimum and maximum ages of patients with CD were 5 and 78 years, respectively, with a mean age of 29.1 ± 16.6 years. In a study conducted in Hamedan, an eastern province of Iran, in 2021, the minimum and maximum ages of CD patients were 3 and 65 years, respectively, with a mean age of 37.8 years [5]. CD can occur at any age, from infancy to old age [17]. A prospective cohort study conducted in Finland, Germany, the USA, and Sweden in 2019 found that most patients develop CD before the age of 10 [18].

The study indicated that the majority of patients with CD were female. A systematic review and meta-analysis conducted in Iran in 2019, which included 2307 patients, reported prevalence rates of CD in men and women as 4.28% and 7.19%, respectively [19]. CD is generally more common in women, with a male-to-female ratio of 2.1 to 3.1 [17]. The higher prevalence of CD in women may be attributed to factors such as differences in healthcare-seeking behavior and reluctance among some men to undergo invasive diagnostic procedures like duodenal biopsy during endoscopy [6].

The study results indicated that approximately 10% of patients with CD were found to have type 1 diabetes. This finding is consistent with studies that have demonstrated the co-occurrence of diabetes and celiac disease (CD). According to Elfström et al., the global prevalence of CD in patients with diabetes was estimated to be 6% [20]. Similarly, a study conducted in Iran in 2020 reported a prevalence of CD in Iranian patients with type 1 diabetes to be 5% (95% CI 3-7) [21].

A systematic review and meta-analysis published in 2021 examined the prevalence of CD in children with type 1 diabetes and reported varying prevalence rates. The prevalence of CD in these patients ranged from 1.4% to 24.5% in studies that used serological tests for diagnosis and from 1.1% to 16.6% in studies that used intestinal biopsy for diagnosis [22]. Differences in prevalence rates between studies can be attributed to various factors, including the diagnostic methods employed and the geographic location of the study population. The prevalence rates of CD in patients with diabetes in Jordan (2019), Saudi Arabia (2019), and Turkey (2020) have been reported to be 16.6%, 15.9%, and 4.4%, respectively [23–25]. These differences in prevalence rates may be influenced by genetic and environmental factors. Type 1 diabetes mellitus and celiac disease are both autoimmune diseases, and they can co-occur due to shared genetic causes [26].

It was observed that approximately two-thirds of patients in the study had iron-deficiency anemia, which is a common symptom of CD. Iron-deficiency anemia can sometimes be the only manifestation of CD in up to 40% of patients [27]. This condition is considered an extraintestinal symptom of CD and may indicate subclinical disease. It occurs due to impaired iron absorption caused by damage to the intestinal mucosal epithelium and occult gastrointestinal bleeding [28, 29].

The findings of this study are consistent with a study conducted in India in 2018, which reported that 81.5% of patients with CD had iron-deficiency anemia [30]. In a systematic review and meta-analysis involving 2998 patients, the prevalence of CD in patients with iron-deficiency anemia was found to be 3.2% [31]. Similarly, prevalence rates of celiac disease in patients with iron-deficiency anemia were reported as 3.26% in Brazil in 2021 and 11.1% in Argentina in 2017 [28, 29]. These findings highlight the importance of considering celiac disease as a possible underlying cause in cases of nonresponsive iron-deficiency anemia.

The study results showed that approximately 40% of patients had growth retardation, which is a common manifestation of the intestinal form of CD [17]. This finding is consistent with studies conducted in the United States (2018) and Saudi Arabia (2017) that reported growth retardation as a common manifestation in children with CD [32, 33]. Regarding family history, the study demonstrated that the majority of patients had no family history of CD. A study conducted in Turkey (2005) found that out of 60 patients with CD, only one had a positive family history [34]. However, in a study conducted in the United States (2018) with 577 CD patients, 152 reported a family history of CD [33]. The presence or absence of a family history of celiac disease (CD) can vary among different populations.

In terms of BMI, the current study showed that approximately half of the patients had a normal BMI, while one-fifth was overweight and one-third was underweight. A study conducted in Sweden in 2014 found that children with CD were underweight and shorter, with a lower BMI compared to healthy children [35]. However, it is important to note that CD is not always associated with malnutrition and weight loss, and individuals with CD may have normal weight or even be overweight [36]. The relationship between overweight and CD is still not fully understood, and further longitudinal studies are needed to explore this issue.

The study found that approximately 20% of patients had IBS. Prevalence rates of CD in patients with IBS have been reported in various studies, including 6.13% in Iran (2019), 1.6% in Tanzania (2019), and 1.01% in China (2015) [19, 37]. The prevalence of CD in Iranian patients with IBS appears to be higher compared to global estimates [19], which may be influenced by factors such as diet, genetics, and culture.

Nearly half of the patients in the study were deficient in vitamin D3. This finding is consistent with a study conducted in Sweden (2017) that reported vitamin D3 deficiency in 73 CD patients [38]. Vitamin D deficiency is a common issue in patients with celiac disease (CD) and is often associated with malabsorption.

The study data revealed that the most common gastrointestinal manifestations in patients with CD were abdominal pain, bloating, and constipation. These findings align with a study conducted in Hamedan, Iran (2021), which reported abdominal pain and diarrhea as the most common gastrointestinal symptoms in CD patients [5]. Similarly, a study in the United States (2011) found that abdominal pain and diarrhea were the most common gastrointestinal symptoms in CD patients who were normal or overweight [39]. In children under three years of age, common gastrointestinal manifestations of CD include diarrhea, anorexia, bloating, and failure to thrive. In children over the age of three and adults, common gastrointestinal symptoms may include bloating, diarrhea, constipation, abdominal pain, and weight loss [17]. Depending on the age group, patients with CD may experience varying symptoms.

### 4.1. Study Limitation

By 2021, the number of patients with celiac disease had dramatically dropped, possibly due to the COVID-19 pandemic, which may affect the estimation of the overall prevalence of the disease. The effect of diet on the prevalence of celiac disease may limit the generalizability of the results.

## 5. Conclusions

The study determined that the overall prevalence of celiac disease in Kermanshah Province, Western Iran, was 5.49%, which is higher than the global average. The most common gastrointestinal manifestations of the disease were abdominal pain, constipation, and diarrhea. Iron-deficiency anemia and vitamin D3 deficiency were the most common nongastrointestinal manifestations. Considering the association of celiac disease with conditions such as diabetes mellitus, irritable bowel syndrome, growth retardation, and iron-deficiency anemia, screening for celiac disease in patients is recommended. Healthcare providers should consider community-based education regarding the importance of following a proper diet and reducing wheat consumption. Longitudinal studies are recommended for monitoring the health status of patients with celiac disease.

## Figures and Tables

**Table 1:** Demographic and clinical characteristics of patients with celiac disease by age group.

Variables	Total	Child (0-14)	Adult (over 14)
	*n* (%)	*n* (%)	*n* (%)
Gender			
Male	35 (30.9)	10 (43.5)	25 (27.8)
Female	78 (69.1)	13 (56.5)	65 (72.2)
Education			
Under high school diploma	93 (82.3)	22 (95.6)	71 (78.9)
Above high school diploma	20 (17.7)	1 (4.4)	19 (21.1)
Body mass index			
Underweight (<18.4)	34 (30.1)	19 (82.6)	15 (16.7)
Normal (18.5-24.9)	54 (47.8)	4 (17.4)	50 (55.5)
Overweight (25-29.9)	25 (22.1)	0 (0.0)	25 (27.8)
Job			
Unemployed	103 (91.1)	22 (95.6)	81 (90.0)
Employed	10 (8.9)	1 (4.4)	9 (10.0)
Family history of irritable bowel syndrome			
Yes	14 (12.4)	0 (0.0)	14 (15.6)
No	99 (87.6)	23 (100.0)	76 (84.4)
Family history of celiac disease			
Yes	6 (5.3)	1 (4.4)	5 (5.6)
No	107 (94.7)	22 (95.6)	85 (94.4)

**Table 2:** Prevalence of celiac disease in Kermanshah Province, Western Iran, from 2019 to 2021.

Variables	Years of study		
	2019	2020	2021
Population	1952434	2058545	2164656
Total number of patients	26	67	20
Prevalence rate per 100,000	1.33	3.25	0.92
Overall prevalence rate per 100,000 population	5.49% (95% CI: 5.17, 5.82)		

**Table 3:** Frequency distribution of gastrointestinal manifestations in patients with celiac disease.

Symptoms	Total, *n* (%)	Child (0-14), *n* (%)	Adult (over 14), *n* (%)
Abdominal pain			
Yes	88 (77.8)	15 (65.2)	73 (81.1)
No	25 (22.2)	8 (34.8)	17 (18.9)
Constipation			
Yes	55 (48.7)	12 (52.7)	43 (47.8)
No	58 (51.3)	11 (47. 3)	47 (52.2)
Diarrhea			
Yes	62 (54.9)	7 (30.4)	55 (61.1)
No	51 (45.1)	16 (69.6)	35 (38.9)
Nausea			
Yes	46 (40.7)	7 (30.4)	39 (43.3)
No	67 (59.3)	16 (69.6)	51 (56.7)
Vomit			
Yes	12 (10.6)	2 (8.7)	10 (11.1)
No	101 (89.4)	21 (91.3)	80 (88.9)
Anorexia			
Yes	34 (30.1)	9 (39.1)	25 (27.8)
No	79 (69.9)	14 (60.9)	65 (72. 2)
Distention			
Yes	67 (59.3)	13 (56.5)	54 (60.0)
No	46 (40.7)	10 (43.5)	36 (40.0)
Dyspepsia			
Yes	21 (18.6)	3 (13.1)	18 (20.0)
No	92 (81.4)	20 (86.9)	72 (80.0)
Irritable bowel syndrome			
Yes	20 (17.7)	1 (4.4)	19 (21.1)
No	93 (82.3)	22 (95.6)	71 (78.9)

**Table 4:** Frequency distribution of nongastrointestinal manifestations in patients with celiac disease.

Symptoms	Total, *n* (%)	Child (0-14), *n* (%)	Adult (over 14), *n* (%)
Oral problems			
Yes	33 (29.2)	10 (43.4)	23 (25.6)
No	80 (70.8)	13 (56.6)	67 (74.4)
Bone and joint problems			
Yes	50 (44.3)	8 (34.8)	42 (46.7)
No	63 (55.7)	15 (65.2)	48 (53.3)
Vitamin D3 deficiency^£^			
Yes	52 (46.1)	9 (39.3)	43 (47.8)
No	61 (53.9)	14 (60.9)	47 (52.2)
Iron-deficiency anemia			
Yes	73 (64.6)	16 (69.6)	57 (63.3)
No	40 (35.4)	7 (30.4)	33 (36.7)
Irritable bowel syndrome			
Yes	20 (17.7)	1 (4.4)	19 (21.1)
No	93 (82.3)	22 (95.6)	71 (78.9)
Rheumatoid arthritis			
Yes	4 (3.5)	0 (0.0)	4 (4.4)
No	109 (96.5)	23 (100.0)	86 (95.6)
Migraine headache			
Yes	11 (9.7)	2 (8.7)	9 (10.0)
No	102 (90.3)	21 (91.3)	81 (90.0)
Sjogren's syndrome			
Yes	13 (11.5)	2 (8.7)	11 (12.2)
No	100 (88.5)	21 (91.3)	79 (87.8)
Hypothyroidism			
Yes	16 (14.2)	2 (8.7)	14 (15.6)
No	97 (85.8)	21 (91.3)	76 (84.4)
Diabetes mellitus			
Yes	13 (11.5)	8 (34.8)	5 (5.6)
No	100 (88.5)	15 (65.2)	85 (94.4)
Growth retardation			
Yes	44 (38.9)	6 (26.1)	38 (42.2)
No	69 (61.1)	17 (73.9)	52 (57.8)

^£^Severe deficiency, moderate deficiency, and insufficiency.

## Data Availability

The identified datasets analyzed during the current study are available from the corresponding author upon reasonable request.
